# Temporal Lobe and Frontal-Subcortical Dissociations in Non-Demented Parkinson’s Disease with Verbal Memory Impairment

**DOI:** 10.1371/journal.pone.0133792

**Published:** 2015-07-24

**Authors:** Jared J. Tanner, Thomas H. Mareci, Michael S. Okun, Dawn Bowers, David J. Libon, Catherine C. Price

**Affiliations:** 1 Department of Clinical and Health Psychology, University of Florida, Gainesville, Florida, United States of America; 2 Department of Biochemistry and Molecular Biology, University of Florida, Gainesville, Florida, United States of America; 3 Department of Neurology, UF Center for Movement Disorders and Neurorestoration, University of Florida, Gainesville, Florida, United States of America; 4 Drexel Neuroscience Institute, Drexel University, College of Medicine, Philadelphia, Pennsylvania, United States of America; Nathan Kline Institute and New York University School of Medicine, UNITED STATES

## Abstract

**Objective:**

The current investigation examined verbal memory in idiopathic non-dementia Parkinson’s disease and the significance of the left entorhinal cortex and left entorhinal-retrosplenial region connections (via temporal cingulum) on memory impairment in Parkinson’s disease.

**Methods:**

Forty non-demented Parkinson’s disease patients and forty non-Parkinson’s disease controls completed two verbal memory tests – a wordlist measure (Philadelphia repeatable Verbal Memory Test) and a story measure (Logical Memory). All participants received T1-weighted and diffusion magnetic resonance imaging (3T; Siemens) sequences. Left entorhinal volume and left entorhinal-retrosplenial connectivity (temporal cingulum edge weight) were the primary imaging variables of interest with frontal lobe thickness and subcortical structure volumes as dissociating variables.

**Results:**

Individuals with Parkinson’s disease showed worse verbal memory, smaller entorhinal volumes, but did not differ in entorhinal-retrosplenial connectivity. For Parkinson’s disease entorhinal-retrosplenial edge weight had the strongest associations with verbal memory. A subset of Parkinson’s disease patients (23%) had deficits (z-scores < -1.5) across both memory measures. Relative to non-impaired Parkinson’s peers, this memory-impaired group had smaller entorhinal volumes.

**Discussion:**

Although entorhinal cortex volume was significantly reduced in Parkinson’s disease patients relative to non-Parkinson’s peers, only white matter connections associated with the entorhinal cortex were significantly associated with verbal memory performance in our sample. There was also no suggestion of contribution from frontal-subcortical gray or frontal white matter regions. These findings argue for additional investigation into medial temporal lobe gray and white matter connectivity for understanding memory in Parkinson’s disease.

## Introduction

Individuals with Parkinson’s disease (PD) frequently self-report problems with memory (e.g., most often reporting difficulty recalling names or words, etc.). Some individuals with PD and mild cognitive impairment or dementia have elements of anterograde memory difficulties commonly associated with entorhinal/hippocampal disruption[1–3]. The entorhinal cortex (ERC) is affected by PD pathology and atrophy in PD[3]. Alpha-synuclein and amyloid-beta have been confirmed in the ERC of non-demented PD and increased pathological burden in PD patients with dementia[4]. Since pathology and cell loss affects medial temporal lobe (MTL) structures in PD[3], white matter networks connecting to the MTL are worthy of consideration.

*In vivo* diffusion imaging shows convincing evidence of reduced white matter integrity in the temporal lobes of cognitively impaired individuals with PD[5–7]. In light of evidence for temporal lobe gray matter and white matter changes in PD, analyses of specific white matter tracts connecting the ERC to limbic structures are needed. The temporal lobe white matter connections between the ERC and retrosplenial cortex (RSC) are presumed to play a role in anterograde memory formation. Up to 20% of the connections to the entorhinal cortex in monkeys are from the retrosplenial cortex with similar connectivity hypothesized in humans[8]. Damage anywhere along this circuit–ERC, ERC-RSC connections, or retrosplenial/posterior cingulate–results in a ‘disconnection’ of MTL structures from the broader limbic region and other association cortices[9].

The current study prospectively recruited individuals with PD relative to matched non-PD peers. The primary goal of the current research was to assess the relative contribution of ERC and associated connections to the RSC on verbal memory function in PD versus that of non-PD peers. Temporal cingulum connectivity was quantified by edge weight, a metric based on graph theory[10].

We hypothesized that ERC cortices and associated white matter connections between ERC and the RSC would be reduced in PD relative to non-PD peers, and that a PD subgroup demonstrating significant memory deficits on verbal memory testing (PD-MI) would show reduced temporal lobe structures relative to both non-memory impaired PD peers (PD-Well) and non-PD peers. To rule out the contribution of frontal-subcortical regions on verbal working memory, we examined the contribution of total caudate nucleus, prefrontal white matter, and frontal thickness, with secondary areas of interest including the putamen and thalamus. These areas were additionally compared between PD-MI and PD-Well individuals.

## Methods

### Participants

The study was approved by the University of Florida Health Center Institutional Review Board (Protocol #472–2007). Providers at the UF Center for Movement Disorder and Neurorestoration referred only non-demented individuals with PD. Structured telephone screening was also performed to verify PD and non-PD participants had intact general cognition. Only non-demented participants who had capacity to consent to participation were included in the study. Written consent was obtained from all participants and all research followed the Declaration of Helsinki. Neurocognitive status was confirmed through neurocognitive screening (Dementia Rating Scale – 2 (DRS-2;[11]) with a total DRS-2 score ≥ 130) and comprehensive neuropsychological assessment overseen by a licensed neuropsychologist (C.P.) as part of the current study. Inclusion Criteria: Right-handed[12], Dementia Rating Scale-Revised (DRS-2[12] raw score >130) and fluent English. PD required diagnosis by movement disorder neurologist, UK Parkinson’s Disease Society Brain Bank Clinical Diagnostic Criteria[13] and Hoehn and Yahr scale[14] ranging from 1–3. Medical Exclusions included: diseases likely to confound cognition (e.g., cerebrovascular accident in the last six months, etc.), deep brain stimulation, secondary/atypical Parkinsonism, and major psychiatric disorder. Depression and apathy were not exclusion criteria due to their high prevalence in PD.

### Procedures

While on medication, participants completed cognitive testing and neuroimaging in order to acquire optimal performance and represent typical participant functioning. All participants also completed measures of general cognition and mood, PD symptoms and severity, comorbidity[15], and a neuropsychological protocol. Medications were reverted to a common metric (Levodopa Equivalency Dose; LED[16]). Raters blinded to group diagnosis double scored and entered all data.

#### Verbal Memory Measures

Due to hypothesized sensitivity to medial temporal lobe function, long delay free recall, long delay savings, and recognition index scores were acquired from two separate verbal memory measures. Two measures were chosen due to convincing arguments that performance may depend on test type. It has been recommended that both list-learning and story memory tests be included for examining declarative memory in PD[17].

*Philadelphia (repeatable) Verbal Learning Test (P(r)VLT)*–is a verbal serial list learning test that was designed specifically for older adults. The P(r)VLT 9-word test is cited in our earlier dementia work[18], with the P(r)VLT 12-word test cited in MCI literature[19]. Dependent variables (DVs) were raw metrics of the following indices: long delay free recall (LDFR total), delay savings (LDFR/immediate free recall Trial 5), and recognition discriminability calculated from signal detection theory (calculated in SPSS as PROBIT(Hit Rate)–PROBIT(Miss Rate); Hit Rate = # correct words recognized/12 and miss rate is # of incorrect words/36; where hit rate = 1, rate was corrected to 1–(1/(2*12)); where miss rate = 0, rate was adjusted to 1/(2*36))[20].

*Wechsler Memory Scale 3rd Revision (WMS-III) Logical Memory*[21] – is a paragraph recall test where the participant listens to two stories, recalls this information from memory both immediately after they have heard each story and again after a thirty minute filled delay, as well as yes/no answers to recognition questions. DVs included raw scores for delay recall, savings (delay/immediate recall), and recognition discriminability (calculated in SPSS as PROBIT(Hit Rate)–PROBIT(Miss Rate): Hit Rate = # correct/30, Miss Rate = # incorrect/30; where hit rate = 1, adjusted to 1-(1/(2*30)); where miss rate = 0, adjusted to 1/(2*30)).

A verbal memory composite score was created from the index scores of both the P(r)VLT and LM, with the non-PD group serving as the normative reference.

#### Background Measures of Processing Speed and Working Memory

To assess whether processing speed and working memory significantly contributed to verbal memory performances in the Non-PD and PD individuals, standardized composites were created from published norms[21–23].

*Processing Speed*–calculated as a composite of the Trail Making Test- Part A (total time), WAIS-III Digit Symbol (total correct) and Stroop Color Word Test–Word Reading condition (total correct).

*Working Memory*–Wechsler Memory Scale-III Digit Span Backward (total span), Spatial Span Backward (total score), and Letter Number Sequencing (total correct).

### Classification of PD Memory Impaired Subgroup (PD-MI)

In light of comments from the 2012 Movement Disorder Society task force on cognitive impairment in PD[24] and MCI classification for dementia[25] individuals were classified using a conservative definition as PD Memory Impaired (PD-MI) if the verbal memory composite score was z≤-1.5. Individuals scoring above this cutoff were classified as PD-Well (PD-Well) for group comparisons on anatomical metrics.

### MRI Protocol

Data were acquired with a Siemens 3T Verio using an 8-channel head coil. We acquired two T1-weighted scans (176 contiguous slices, 1mm^3^ voxels, TR/TE = 2500/3.77ms) for gray analyses, T2-weighted images (176 contiguous slices, 1mm^3^ voxels, TR/TE = 3200/409ms) to improve skull segmentation for total intracranial volume (TICV) measurement, and two separate single-shot echo planar imaging (EPI) diffusion weighted images with gradients applied along 6 directions (diffusion weight = 100s/mm^2^) and 64 directions (diffusion weight = 1000s/mm^2^) for tractography. Diffusion imaging parameters were set at 73 contiguous axial slices with 2mm^3^ voxels and TR/TE = 17300/81ms. Motion correction, cortical reconstruction, and volumetric segmentation were completed with FreeSurfer 5.3; all data were quality checked. Segmentation procedural and technical details are described elsewhere[26,27].

#### Neuroanatomical Regions of Interest for Volumetrics, Thickness, and Diffusion Analyses

*ERCs* were manually traced in ITK-SNAP[28] by an expert rater (intra-rater Dice Similarity Coefficient (DSC)>0.8, n = 20) blinded to diagnosis. Briefly, each motion-corrected brain was aligned to the MNI152 template provided with the FMRIB Software Library (FSL) using a rigid body via FMRIB’s Linear Image Registration Tool (FLIRT)[29]. Measurement then followed published procedures[8,30]. The final variable of interest was left entorhinal volume in mm^3^.

*RSC* was created by exporting the gray-white boundary of the isthmus of the cingulate from FreeSurfer[31], inflating by 1mm in 3 dimensions, and importing into ITK-SNAP for manual cleaning by an expert, blinded rater to localize the region of interest (ROI) to the retrosplenial region. Reliability was high (intra-rater DSC>0.80, n = 20). The final region of interest was constrained to Brodmann areas 29 and 30[32] and up to 4mm of surrounding non-callosal matter to track the temporal cingulum; thus, the final ROI included portions of cingulate area 23 and underlying white matter.

*Frontal lobe thickness*, *caudate*, *putamen*, *and thalamus volumes* were acquired from FreeSurfer and checked for accuracy. The final variables of interest were mean bilateral frontal thickness and total bilateral caudate, putamen, and thalamus volumes to represent frontal-subcortical integrity.

*Prefrontal and Temporal FA* values were calculated by rigid body transforming prefrontal and temporal white matter masks into diffusion space and overlaying individual FA maps to determine regional values. Prefrontal FA involved the region anterior to the rostrum of the corpus callosum and temporal FA involved all white matter within the boundaries of the temporal lobe. Final variables of interest were mean left prefrontal and temporal white matter FA.

*Control variable*: *Intracranial volume* masks were estimated using FSL Brain Extraction Tool (BET) version 2.1[33] and then manually cleaned by expert raters to fill the entire space within the inner surface of the skull superior to a straight line between the occipital bone and the clivus. Reliability was high (DSC>0.99). The final variable of interest was TICV in mm^3^.

#### Diffusion Processing

Diffusion data were preprocessed using freely available in-house software written in IDL (Exelis Visual Information Solutions, Boulder, CO). Eddy current correction was performed using FSL. Diffusion tensor imaging metrics (FA) were calculated using FSL. Participant motion during diffusion sequences was quantified using TRACULA[34]. For fiber tracking, fiber orientation profiles were estimated based on the mixture of Wishart method outlined by Jian and colleagues[35]. Diffusion images were trilinearly interpolated[29] to 1mm^3^ voxels and whole brain deterministic fiber tracking initiated using 125 uniformly-distributed streamlines per voxel.

ERC and RSC ROIs were linearly transformed into interpolated diffusion space for fiber tracking between them. Connectivity strength between ROIs was obtained by calculating the normalized edge weight, *w*(*e*_*ij*_ *)*, a graph metric described in detail elsewhere[10], with an additional adjustment for TICV to minimize the effects of head size on connectivity metrics[36]; edge weight controls for ROI surface area. The final variable of interest was ERC-RSC *w(e*_*ij*_ *)*. To investigate frontal and temporal lobe white matter changes, tract-based spatial statistics (TBSS) was run using published procedures[37] with a group-specific FA template used as registration target and number of permutations set at 20,000.

### Statistical Analyses

Statistical analyses were performed using SPSS 22.0. Non-Gaussian distributions of cognitive and neuroimaging data were adjusted using power transformations[38]. Multivariate analysis of variance (MANOVA) assessed between group verbal memory performance by test (P(r)VLT, Logical Memory) with processing speed and working memory composites examined separately as potential covariates.

Quantifications of the ERC, and temporal cingulum *w*(*e*_*ij*_ *)* were entered into *t*-test analyses to determine group (PD vs. non-PD) differences. For PD-MI Memory Impaired (PD MI) relative to PD-Well, an additional *t*-test compared left ERC volume, left temporal and frontal cortex thickness, left ERC-RSC *w(e*_*ij*_ *)*, left prefrontal and temporal FA, and bilateral caudate, putamen, and thalamus volumes. These brain variables were also entered into hierarchical regression models to predict the verbal memory composite score. For structure-cognition dissociations, working memory and inhibition composite scores were alternatively entered into regression models.

## Results

### Demographics

Table 1. From 186 individuals screened by telephone, 43 individuals with PD and 41 non-PD peers met criteria. Four enrolled participants could not complete MRI (i.e., claustrophobia, metal artifact). Final sample involved 40 PD and 40 non-PD. Groups were statistically similar for general demographics, comorbidity, premorbid intellect estimates and general cognition estimates. All were independent in instrumental activities of daily living with all but one PD individual independently managing medications. PD was largely unilateral tremor dominant (70% H&Y≤1.5).

**Table 1:** Parkinson’s Disease (PD) and Non-PD “Control” Demographics, General Cognition, and Disease Metrics.

Measure	PD (n = 40)	Control (n = 40)	T	P Value
Age	67.80±5.44, 60/79	68.18±4.64, 62/79	0.33	0.74
Sex (M:F)	32:8	33:7	-0.28	0.78
Education	16.28±3.03, 10/22	16.75±2.35, 12/20	0.78	0.44
DRS-2 Total	139.43±3.13, 131/144	140.20±2.49, 133/144	1.23	0.22
WTAR	107.35±7.68, 86/118	108.80±8.76, 81/119	0.79	0.43
Comorbidity	0.30±0.72, 0/4	0.28±0.61, 0/2	-0.12	0.91
UPDRS Part 3	17.60±10.73, 3/46	2.75±3.36, 0/15	-8.36	0.00
Disease (yrs)	7.50±5.15, 1/26	—	—	—
l-Dopa Eq.	685.69±371.49, 0/1450	1.00±6.32, 0/40	-11.66	0.00

DRS-2 = Dementia Rating Scale – 2^nd^ Version total raw score (max = 144); WTAR = Wechsler Test of Adult Reading raw score; UPDRS Part 3 = United Parkinson’s Disease Rating Scale Part 3 (Motor) score; Disease duration (yrs) = years of disease duration per self-report/medical records; l-Dopa Equiv. Score = Levodopa Equivalent Score (total daily levodopa dosage intake in milligrams). One control was on levodopa for restless leg syndrome.

### PD vs. Control Verbal Memory Performance

Table 2. For the P(r)VLT, PD scored lower than non-PD controls (F(3,75) = 6.97, p<0.001). This group difference was maintained after covarying for processing speed (F(3,74) = 3.32, p = 0.02) and working memory (F(3,74) = 4.11, p<0.01). Across all three Logical Memory (LM) variables, PD also scored lower than non-PD (F(3,76) = 4.03, p = 0.01). However, for Logical Memory the effect of group was no longer significant after covarying for processing speed (F(3,75) = 1.50, p = 0.22) and was a trend when covarying for working memory (F(3,75) = 2.72, p = 0.05).

**Table 2:** Verbal Memory in Parkinson’s disease (PD) and Non-Parkinson’s disease (Non-PD) Raw Mean, Standard Deviation, and Minimum/Maximum scores.

Measure	PD (n = 40)	Control (n = 40)	P	Eta^2^
P(r)VLT LDFR^a^	8.08±2.13, 4/12	9.65±2.25, 3/12,	<0.01	0.12*
P(r)VLT Savings^a^	84.36± 17.33, 0.40/1.14	88.43±14.43, 0.43/1.11	0.26	0.00
P(r)VLT R. Dis.^a^	2.94±0.66,1.64/3.93	3.41±0.47, 2.32/3.93	<0.01	0.15**
Logical Mem. LDFR	24.62± 7.98, 11/41	28.53±5.67, 18/39	0.01	0.08*
Logical Mem. Savings	81.07±15.60, 47/109	88.03±13.93, 37/118	0.04	0.06*
Logical Memory R. Dis.	2.20±0.94, 0.15/4.26	2.86±0.84, 1.46/4.26	<0.01	0.13*

^a^P(r)VLT PD sample = 39 due to test exclusion from administration error; all other n = 40 per group.

*Medium effect size

**Large effect size.

LDFR = long delay free recall; R. Discr. = recognition discriminability. Analyses shown in raw form, uncorrected for processing speed or working memory as covariates.

### PD vs. Control ERC and Temporal Cingulum Connectivity

TICV was larger in PD than non-PD (7.7% difference, p<0.01) and served as a neuroanatomical covariate for subcortical gray, cortical gray, and tractography edge weight metrics. Between-group registration and intensity-based metrics[34], demonstrated no significant group differences in diffusion sequence motion (Registration: average translation: t = 0.98, p = 0.33; average rotation: χ^2^ = 1.25, p = 0.26; Intensity: Percent bad slices χ^2^ = 0.26, p = 0.61; Average dropout score χ^2^ = 0.26, p = 0.61).

#### Gray Matter–Left Entorhinal Cortex

Correcting for TICV, individuals with PD had 11% less left ERC volume compared to non-PD (t = 2.71, p<0.01; Cohen’s d = 3.82).

#### White Matter–ERC-RSC and FA

**Fig 1:**
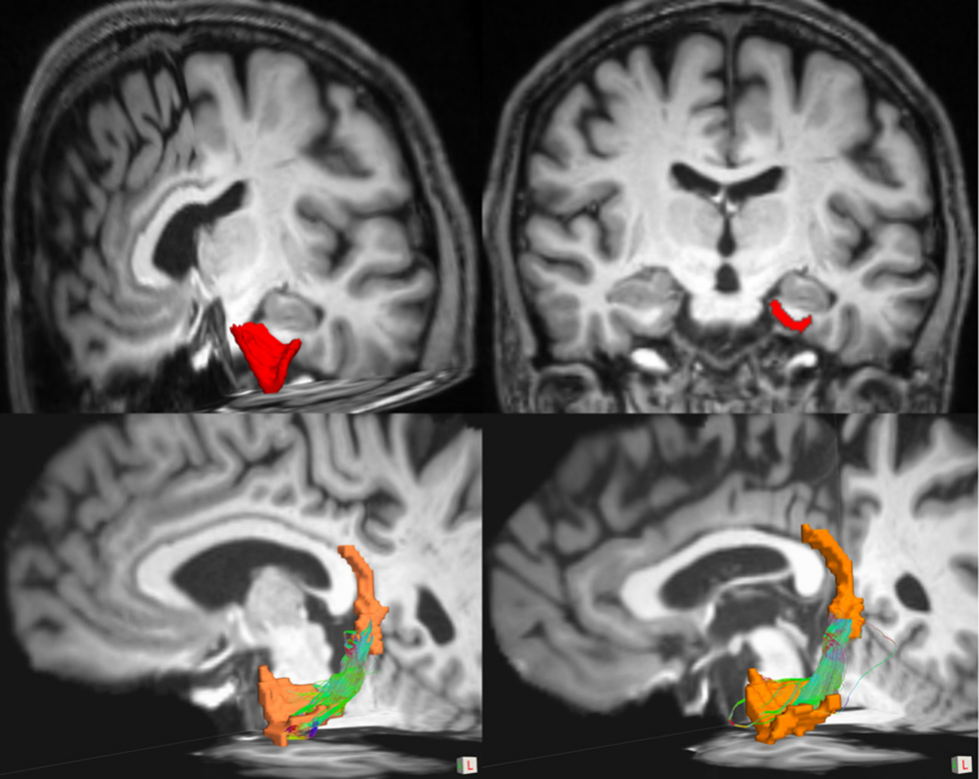
Images representative of entorhinal volumetric and fiber tracking results. Image demonstrating left entorhinal (in red on top panel) and ERC-RSC fibers (temporal cingulum) with entorhinal cortex and retrosplenial region ROIs in orange (bottom panel).

Fig 1. Temporal cingulum *w*(*e*_*ij*_ *)* and temporal FA were statistically similar between PD and non-PD peers (PD temporal cingulum *w*(*e*_*ij*_ *)*: mean = 1.0 x 10^−8^ ± 0.7 x 10^−8^; control temporal cingulum *w*(*e*_*ij*_ *)*: mean = 0.9 x 10^−8^ ± 0.6 x 10^−8^. t = 0.98; p = 0.33; Cohen’s d = 0.22); temporal FA (PD = 0.31±0.02; control FA = 0.30±0.03, p = 0.65; Cohen’s d = 0.39). There were no relationships between disease severity/duration and temporal white matter metrics.

### PD Cognitively Well (PD-Well) versus PD Memory Impaired (PD-MI) Comparisons

Means and standard deviations from the controls were used to identify individuals scoring 1.5 below expectations. Within PD, PD-Well (n = 31) and PD-MI (n = 9) are statistically similar for disease severity/duration and all demographics except education, with the PD-MI having on average two fewer years of education (p = 0.03).

#### Verbal Memory Indices

Separate standardized P(r)VLT and Logical Memory composites were created based on the control group means and standard deviations. Significant group differences remained even after correcting for education: P(r)VLT composite (mean±s.d. PD-Well = -0.35±0.90; PD-MI = -1.70±0.56; F(1,36) = 18.51, p<0.001); Logical Memory composite (mean ±s.d. PD-Well = -0.32±0.89; PD-MI = -1.82±0.58; F(1,36) = 16.57, p<0.001). Group differences remained after controlling for processing speed and working memory (p values≤0.001). Further, PD-MI had lower scores than PD-Well on all six individual index scores (all p values ≤ 0.01), even after controlling for processing speed and working memory (p values ≤ 0.014).

#### Temporal Lobe White and Gray

**Fig 2:**
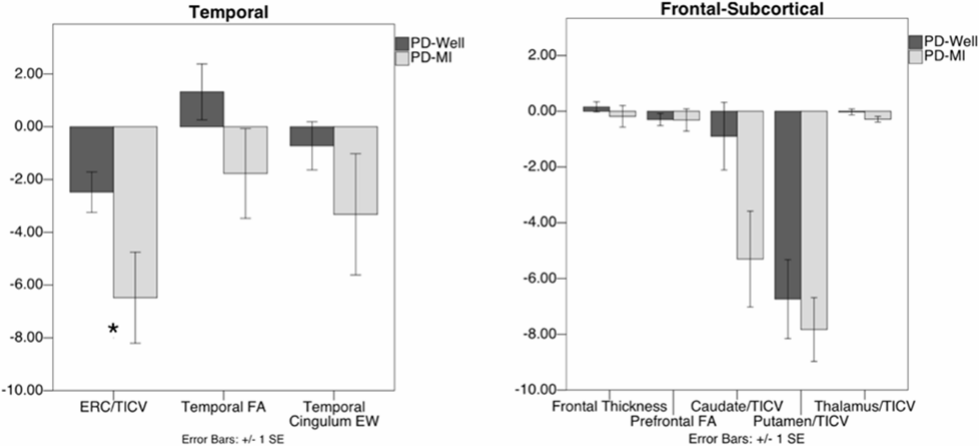
Control-based z scores of temporal and frontal-subcortical regions for PD-Well and PD-MI. *Denotes group difference p<0.05. ERC/TICV = Left entorhinal volume/TICV, Temporal FA = Mean fractional anisotropy of the white matter of the left temporal lobe, Temporal Cingulum EW = Edge weight connectivity of the left temporal cingulum, Frontal thickness = Total mean frontal cortex thickness, Prefrontal FA = Total mean fractional anisotropy of frontal white matter anterior to the rostrum of the corpus callosum, Caudate/TICV = total caudate/TICV, Putamen/TICV = total putamen/TICV, Thalamus/TICV = total thalamus/TICV.

Fig 2. For temporal gray, PD-MI have significantly smaller ERC (p = 0.04). White matter metrics showed no statistical differences but moderate effect sizes for both ERC-RSC *w*(*e*_*ij*_ *)* (p = 0.18; Cohen’s d = 0.48) and temporal FA (p = 0.08, Cohen’s d = 0.65).

#### Frontal-Subcortical Regions of Interest

For gray matter, groups were statistically similar in total frontal cortex thickness (t(38) = 0.86, p = 0.39), putamen (t(38) = 0.40, p = 0.69), and thalamus (t(38) = 1.27, p = 0.21), but with a trend for smaller bilateral caudate nuclei volume (t(38) = 1.81, p = 0.08; Cohen’s d = 0.73) in the PD-MI group. Prefrontal FA was statistically similar overall (t(38) = 0.04, p = 0.97) with this confirmed by tract-based spatial statistics.

### ERC and ERC-RSC Predictors for Verbal Memory in PD

Based on our *a priori* hypotheses, we ran linear regression analyses to examine the relative contribution of the entorhinal cortex versus ERC-RSC on the verbal memory composite. A review of potential covariates (age, UPDRS, disease duration) showed that only age trended to negatively contribute to the verbal memory composite (age: r = -0.29, p = 0.07; education: r = 0.24, p = 0.13; disease duration r = 0.12, p = 0.46; UPDRS-3 r = -0.14, p = 0.40). For this reason, age was entered as a control variable in Block 1 of the hierarchical regression. Block 2: Addition of the left ERC/TICV did not explain additional variance (R = 0.34, R^2^ = 0.10; R^2^ change = 0.03, p = 0.26; beta = 0.17). Block 3: Addition of the ERC-RSC significantly explained more variance (R = 0.47, R^2^ = 0.23, R^2^ change = 0.11, p = 0.03; beta = 0.31). Addition of subcortical structures did not contribute significantly (e.g., caudate: beta = 0.12, p = 0.43). Replacing the ERC-RSC connectivity with temporal FA reduced the significance of the model (Temporal FA beta = 0.18, p = 0.29).

For PD participants, a follow-up index score hierarchical regression with age entered in the first block revealed that ERC volume accounted for an additional 10% of variance in Logical Memory savings score: R = 0.37, R^2^ = 0.14, beta = 0.31, p = 0.05.

### ERC and ERC-RSC Predictors for Verbal Memory in non-PD

For non-PD, as above, age was entered as a control variable in Block 1 of a hierarchical regression (F = 6.62, p = 0.01). Block 2: Addition of the left ERC/TICV did not explain additional variance (R = 0.39, R^2^ = 0.15; R^2^ change = 0.00, p = 0.90; beta = 0.02). Block 3: Addition of the ERC-RSC also did not significantly explain more variance (R = 0.39, R^2^ = 0.16, R^2^ change = 0.01, p = 0.60; beta = 0.08). Addition of subcortical structures did not contribute significantly (e.g., caudate: beta = -0.18, p = 0.37). Replacing the ERC-RSC connectivity with temporal FA did not improve the model (Temporal FA beta = -0.04, p = 0.82).

For the non-PD, follow-up index score hierarchical regressions with age entered as first block revealed that ERC volume accounted for 17% of variance in Logical Memory savings score: R = 0.41, R^2^ = 0.17, beta = 0.40, p = 0.01.

## Discussion

For our sample of idiopathic tremor dominant non-demented PD, recall and retention of new verbal information was significantly impaired relative to non-PD control peers. In PD, memory performance was best explained by the left ERC-RSC white matter connection and not the left ERC alone. This is a significant finding. Although the ERC was significantly reduced in the PD relative to non-PD peers, only the white matter connections associated with the ERC were significantly associated with verbal memory performance in our sample. While PD pathology might initially affect the entorhinal regions, the white matter disruption stemming from these key regions seems particularly relevant for verbal memory performance in our non-dementia PD participants.

Our findings extend upon recent investigations discussing verbal memory performance in PD. Multiple studies have demonstrated varying levels of anterograde memory deficits in PD[1,3,39]. However, previous studies used only one memory measure to classify deficits and few [1,3,39] have neuroimaging to confirm medial temporal lobe structure. The rate of anterograde deficits in our sample (23%) replicates the percent previously reported[1]. We uniquely show, however, that memory abilities in PD were explained best by MTL structures of interest–not frontal-subcortical structures/frontal white matter. Further, our prospective use of memory, executive function, and processing speed measures clarified that our samples’ learning/memory abilities could not be fully explained by processing speed. Declarative memory impairment in non-demented individuals with PD historically was believed to result from executive and processing speed deficits dysfunction in frontal-subcortical loops[40]. Although there was evidence of processing speed associations with one memory metric (LM), the association did not hold across the combined metric of the list learning and story memory tasks. Additionally, processing speed scores did not significantly explain memory performance differences between the PD-MI and PD-Well individuals and there was no suggestion of contribution from frontal-subcortical gray or frontal white matter in the PD-MI subgroup. Rather, the entorhinal cortex and associated connections to the retrosplenial region explained memory test performance. These collective findings suggest that the memory impairment in PD cannot be fully explained on the basis of ‘dysexecutive impairment; that is, on the basis of a retrieval-based model presumably mediated by frontal-subcortical involvement[18]. Lack of consistency on this issue as reflected in the literature[17,39–41] could be related to heterogeneity in PD regarding both memory and learning abilities and underlying neuroanatomy. Such heterogeneity has been shown to be present in other dementia and dementia-related syndromes[42] and should be the subject of further research in PD. The lack of consistency in research assessing verbal memory in PD is also partially caused by differences in research design.

Regarding ERC quantification, there were group differences in ERC volume for the PD relative to non-PD controls (11% difference in mean volumes), as well as PD-MI relative to PD-Well (and non-PD peers). Although ERC did not explain, over that of age, the verbal memory composite score, post-hoc analyses showed that ERC volume associated with Logical Memory savings (retention) such that larger volumes associated with less ‘forgetting’ of the stories. These findings allow us to argue that while the ERC metrics are valid, they are not the primary source of memory disruption across the two verbal memory tests in our non-demented sample. Interestingly, although the ERC volumes for non-dementia PD and non-PD are similar to the non-dementia PD participants reported in Goldman et al.[3], only Goldman and colleagues identified a relationship between ERC volumes and memory metrics. We speculate that this study difference may be due to Goldman and colleagues’ additional inclusion of dementia PD within their full analysis; our focus on PD non-dementia may have restricted ERC-memory function analytical range.

Only white matter connections from the ERC to the retrosplenial region contributed to memory performance in our non-dementia PD, independently explaining 11% of variance in delayed memory across both memory measures. The retrosplenial cortex is a major termination site for the outflow of acetylcholine from the basal forebrain via the cingulum[43], and thus plays a major role in memory function. Importantly, our measure of white matter integrity is dependent upon gray matter surface area. Mathematically, only fibers terminating within each region of interest were included in the edge weight calculation. Edge weight as calculated for this study therefore represents both white and gray matter, capturing the integrity of a network without being limited to individual components of a network. This likely explains why within PD, ERC-RSC edge weight was a sensitive predictor of memory performance while ERC volume by itself was not.

There is accumulating research regarding the importance of temporal lobe white matter on memory function. White matter integrity near the entorhinal cortex and in the left inferior longitudinal fasciculus is relevant to memory function in temporal lobe epilepsy[44]. In a diffusion tensor analysis of white matter in PD, researchers reported verbal memory associations with mean diffusivity in the fornix and anterior corona radiata[45]. Additional research investigating gray and white matter contributions to cognition is needed. Memory functions rely on widespread networks including the Papez circuit and other frontal-temporal connections. With technological advances and growing appreciation of connectivity[46], it is no longer sufficient to examine individual gray matter structure or white matter structure alone.

This study requires replication, particularly for the association between ERC-RSC edge weight and verbal memory in PD. It is recognized that the study participants are not fully representative of a broad population and therefore the findings might not generalize to the entire PD population or other memory forms (e.g., visual based memory measures). The sample also had restricted variability in verbal memory scores which appear indicative of the high general cognitive functioning of the individuals within the sample. Further, while the overall sample sizes were adequate, the PD-MI group only included nine individuals, potentially limiting the generalizability of findings to the broader PD population. We also recognize that classifying PD-MI to include those who averaged 1.5 standard deviations below peers on two memory tests is more a stringent classification for MCI than has been proposed being used for broader MCI analyses[25,47] but it follows the guidelines for classifying cognitive impairment as specified by the Movement Disorder Society Task Force[48].

Another limitation is the inherent challenges in mapping fiber networks using MRI[49]. Steps were taken, however, during processing to minimize error. Tracking results also match known anatomy. Future directions include replicating results with a larger sample and assessing gray-white matter interactions supporting visual memory performance in PD.

In conclusion, this study demonstrated that 23% of individuals with PD experience poor encoding, storage, and retrieval of verbal information. ERC-RSC connectivity, not ERC volume alone or frontal-subcortical regions of interest, contributed to memory performance. This study argues for the consideration of interactions between gray and white regions of interest for cognitive decline before dementia in PD.

## References

[1] Filoteo J, Rilling L, Cole B, Williams B, Davis J, Roberts J. Variable memory profiles in Parkinson's disease. J Clin Exp Neuropsychol. 1997;19: 878–888. 9524882 10.1080/01688639708403768

[2] Tröster AI. A Précis of Recent Advances in the Neuropsychology of Mild Cognitive Impairment(s) in Parkinson's Disease and a Proposal of Preliminary Research Criteria. J Int Neuropsychol Soc. 2011;17: 393–406. 10.1017/S1355617711000257 21473805

[3] Goldman JG, Stebbins GT, Bernard B, Stoub TR, Goetz CG, deToledo Morrell L. Entorhinal cortex atrophy differentiates Parkinson's disease patients with and without dementia. Mov Disord. 2012;27: 727–734. 10.1002/mds.24938 22410753 PMC3366041

[4] Kalaitzakis ME, Christian LM, Moran LB, Graeber MB, Pearce RKB, Gentleman SM. Dementia and visual hallucinations associated with limbic pathology in Parkinson's disease. Parkinsonism Relat Disord. 2009;15: 196–204. 10.1016/j.parkreldis.2008.05.007 18602855

[5] Hattori T, Orimo S, Aoki S, Ito K, Abe O, Amano A, et al Cognitive status correlates with white matter alteration in Parkinson's disease. Hum Brain Mapp. 2012;33: 727–739. 10.1002/hbm.21245 21495116 PMC6870034

[6] Price CC. Controversial topics in neuroimaging: PD as a disconnection syndrome? JINS. 2012;18: 200.22300634

[7] Zhan W, Kang GA, Glass GA, Zhang Y, Shirley C, Millin R, et al Regional alterations of brain microstructure in Parkinson's disease using diffusion tensor imaging. Mov Disord. 2012;27: 90–97. 10.1002/mds.23917 21850668 PMC4472452

[8] Insausti R, Juottonen K, Soininen H, Insausti A, Partanen K, Vainio P, et al MR volumetric analysis of the human entorhinal, perirhinal, and temporopolar cortices. American Journal of Neuroradiology. 1998;19: 659–671. 9576651 PMC8337393

[9] Salat DH, Tuch DS, van der Kouwe AJW, Greve DN, Pappu V, Lee SY, et al White matter pathology isolates the hippocampal formation in Alzheimer's disease. Neurobiol Aging. 2010;31: 244–256. 10.1016/j.neurobiolaging.2008.03.013 18455835 PMC3038572

[10] Hagmann P, Kurant M, Gigandet X, Thiran P, Wedeen V, Meuli R, et al Mapping human whole-brain structural networks with diffusion MRI. PLoS ONE. 2007;2: 1–9. 10.1371/journal.pone.0000597 PMC1895920 17611629

[11] Matteau E, Dupré N, Langlois M, Jean L, Thivierge S, Provencher P, et al Mattis Dementia Rating Scale 2: screening for MCI and dementia. American Journal of Alzheimer's Disease and Other Dementias. 2011;26: 389–398. 10.1177/1533317511412046 21697143 PMC10845364

[12] Briggs GG, Nebes RD. Patterns of Hand Preference in a Student Population. Cortex. 11: 230–238. 1204363 10.1016/s0010-9452(75)80005-0

[13] Hughes AJ, Ben-Shlomo Y, Daniel SE, Lees AJ. What features improve the accuracy of clinical diagnosis in Parkinson's disease: a clinicopathologic study. Neurology. 1992;42: 1142–1146. 1603339 10.1212/wnl.42.6.1142

[14] Hoehn MM, Yahr MD. Parkinsonism onset, progression, and mortality. Neurology. 1967;17: 427–427. 10.1212/WNL.17.5.427 6067254

[15] Charlson ME, Pompei P, Ales KL, MacKenzie CR. A new method of classifying prognostic comorbidity in longitudinal studies: development and validation. J Chronic Dis. 1987;40: 373–383. 3558716 10.1016/0021-9681(87)90171-8

[16] Tomlinson CL, Stowe R, Patel S, Rick C, Gray R, Clarke CE. Systematic review of levodopa dose equivalency reporting in Parkinson's disease. Mov Disord. 2010;25: 2649–2653. 10.1002/mds.23429 21069833

[17] Zahodne LB, Bowers D, Price CC, Bauer RM, Nisenzon A, Foote KD, et al The Case for Testing Memory With Both Stories and Word Lists Prior to DBS Surgery for Parkinson's Disease. The Clinical Neuropsychologist. 2011;25: 348–358. 10.1080/13854046.2011.562869 21491347 PMC3077807

[18] Price C, Garrett KD, Jefferson A, Cosentino S, Tanner J, Penney D, et al Leukoaraiosis Severity and List-Learning in Dementia. The Clinical Neuropsychologist. 2009;23: 944–961. 10.1080/13854040802681664 19370451 PMC2866111

[19] Libon DJ, Bondi MW, Price CC, Lamar M, Eppig J, Wambach DM, et al Verbal Serial List Learning in Mild Cognitive Impairment: A Profile Analysis of Interference, Forgetting, and Errors. J Int Neuropsychol Soc. 2011;17: 905–914. 10.1017/S1355617711000944 21880171 PMC3315271

[20] Stanislaw H, Todorov N. Calculation of signal detection theory measures. Behav Res Methods Instrum Comput. 1999;31: 137–149. 10495845 10.3758/bf03207704

[21] Wechsler D. Wechsler Memory Scale—Third Edition III. San Antonio, TX: The Psychological Corporation; 1997.

[22] Heaton RK, Psychological Assessment Resources, Inc. Revised Comprehensive Norms for an Expanded Halstead-Reitan Battery: Demographically Adjusted Neuropsychological Norms for African American and Caucasian Adults, Professional Manual. Psychological Assessment Resources; 2004.

[23] Strauss E, Sherman EMS, Spreen O. A Compendium of Neuropsychological Tests: Administration, Norms, and Commentary. Oxford University Press; 2006.

[24] Litvan I, Aarsland D, Adler CH, Goldman JG, Kulisevsky J, Mollenhauer B, et al MDS Task Force on mild cognitive impairment in Parkinson's disease: critical review of PD-MCI. Mov Disord. 2011;26: 1814–1824. 10.1002/mds.23823 21661055 PMC3181006

[25] Bondi MW, Edmonds EC, Jak AJ, Clark LR, Delano-Wood L, McDonald CR, et al Neuropsychological criteria for mild cognitive impairment improves diagnostic precision, biomarker associations, and progression rates. J Alzheimers Dis. 2014;42: 275–289. 10.3233/JAD-140276 24844687 PMC4133291

[26] Fischl B, Dale AM. Measuring the thickness of the human cerebral cortex from magnetic resonance images. Proc Natl Acad Sci USA. 2000;97: 11050–11055. 10.1073/pnas.200033797 10984517 PMC27146

[27] Fischl B, Salat DH, van der Kouwe AJW, Makris N, Ségonne F, Quinn BT, et al Sequence-independent segmentation of magnetic resonance images. NeuroImage. 2004;23: S69–S84. 10.1016/j.neuroimage.2004.07.016 15501102

[28] Yushkevich PA, Piven J, Hazlett HC, Smith RG, Ho S, Gee JC, et al User-guided 3D active contour segmentation of anatomical structures: significantly improved efficiency and reliability. NeuroImage. 2006;31: 1116–1128. 10.1016/j.neuroimage.2006.01.015 16545965

[29] Jenkinson M, Smith S. A global optimisation method for robust affine registration of brain images. Med Image Anal. 2001;5: 143–156. 11516708 10.1016/s1361-8415(01)00036-6

[30] Price CC, Wood MF, Leonard CM, Towler S, Ward J, Montijo H, et al Entorhinal cortex volume in older adults: Reliability and validity considerations for three published measurement protocols. Journal of the International Neuropsychological Society. 2010;16: 846–855. 10.1017/S135561771000072X 20937164 PMC3070302

[31] Fischl B. FreeSurfer. NeuroImage. 2012;62: 774–781. 10.1016/j.neuroimage.2012.01.021 22248573 PMC3685476

[32] Vogt BA, Absher JR, Bush G. Human retrosplenial cortex: where is it and is it involved in emotion? Trends Neurosci. 2000;23: 195–197. 10782121 10.1016/s0166-2236(00)01579-4

[33] Smith SM, Zhang Y, Jenkinson M, Chen J, Matthews PM, Federico A, et al Accurate, robust, and automated longitudinal and cross-sectional brain change analysis. NeuroImage. 2002;17: 479–489. 12482100 10.1006/nimg.2002.1040

[34] Yendiki A, Koldewyn K, Kakunoori S, Kanwisher N, Fischl B. Spurious group differences due to head motion in a diffusion MRI study. NeuroImage. 2013;88C: 79–90. 10.1016/j.neuroimage.2013.11.027 24269273 PMC4029882

[35] Jian B, Vemuri BC, Ozarslan E, Carney PR, Mareci TH. A novel tensor distribution model for the diffusion-weighted MR signal. NeuroImage. 2007;37: 164–176. 10.1016/j.neuroimage.2007.03.074 17570683 PMC2576290

[36] Yan C, Gong G, Wang J, Wang D, Liu D, Zhu C, et al Sex- and brain size-related small-world structural cortical networks in young adults: a DTI tractography study. Cerebral Cortex. 2011;21: 449–458. 10.1093/cercor/bhq111 20562318

[37] Smith SM, Jenkinson M, Johansen-Berg H, Rueckert D, Nichols TE, Mackay CE, et al Tract-based spatial statistics: voxelwise analysis of multi-subject diffusion data. NeuroImage. 2006;31: 1487–1505. 16624579 10.1016/j.neuroimage.2006.02.024

[38] Osborne JW. Improving your data transformations: Applying the Box-Cox transformation. Practical Assessment, Research & Evaluation. 2010;15: 1–9.

[39] Weintraub D, Moberg PJ, Culbertson WC, Duda JE, Stern MB. Evidence for impaired encoding and retrieval memory profiles in Parkinson disease. Cogn Behav Neurol. 2004;17: 195–200. 15622014

[40] Zgaljardic DJ, Borod JC, Foldi NS, Mattis P. A review of the cognitive and behavioral sequelae of Parkinson's disease: relationship to frontostriatal circuitry. Cogn Behav Neurol. 2003;16: 193–210. 14665819 10.1097/00146965-200312000-00001

[41] Brønnick K, Alves G, Aarsland D, Tysnes O-B, Larsen JP, Norwegian ParkWest Study Group. Verbal memory in drug-naive, newly diagnosed Parkinson's disease. The retrieval deficit hypothesis revisited. Neuropsychology. 2011;25: 114–124. 10.1037/a0020857 20954781

[42] Libon DJ, Drabick DAG, Giovannetti T, Price CC, Bondi MW, Eppig J, et al Neuropsychological syndromes associated with Alzheimer's/vascular dementia: a latent class analysis. J Alzheimers Dis. 2014;42: 999–1014. 10.3233/JAD-132147 25024329

[43] Braak H, Ghebremedhin E, Rüb U, Bratzke H, Del Tredici K. Stages in the development of Parkinson's disease-related pathology. Cell Tissue Res. 2004;318: 121–134. 10.1007/s00441-004-0956-9 15338272

[44] McDonald CR, Leyden KM, Hagler DJ, Kucukboyaci NE, Kemmotsu N, Tecoma ES, et al White matter microstructure complements morphometry for predicting verbal memory in epilepsy. 2014;58: 139–150. 10.1016/j.cortex.2014.05.014 25016097 PMC4188700

[45] Zheng Z, Shemmassian S, Wiljekoon C, Kim W, Bookheimer SY, Pouratian N. DTI correlates of distinct cognitive impairments in Parkinson's disease. 2014;35: 1325–1333. 10.1002/hbm.22256 23417856 PMC3664116

[46] Gratwicke J, Jahanshahi M, Foltynie T. Parkinson's disease dementia: a neural networks perspective. Brain. 2015;138: 1454–1476. 10.1093/brain/awv104 25888551 PMC4614131

[47] Jak AJ, Bangen KJ, Wierenga CE, Delano-Wood L, Corey-Bloom J, Bondi MW. Contributions of neuropsychology and neuroimaging to understanding clinical subtypes of mild cognitive impairment. International review of Neurobiology. 2009;84: 81–103. 10.1016/S0074-7742(09)00405-X 19501714 PMC2864107

[48] Litvan I, Goldman JG, Tröster AI, Schmand BA, Weintraub D, Petersen RC, et al Diagnostic criteria for mild cognitive impairment in Parkinson's disease: Movement Disorder Society Task Force guidelines. Mov Disord. 2012;27: 349–356. 10.1002/mds.24893 22275317 PMC3641655

[49] Jones D. Challenges and limitations of quantifying brain connectivity in vivo with diffusion MRI. Imaging Med. 2010;2: 341–355.

